# *trans*-Bis[2,5-bis­(pyridin-2-yl)-1,3,4-thia­diazole-κ^2^ *N* ^2^,*N* ^3^]bis­(methanol-κ*O*)iron(II) bis­(perchlorate)

**DOI:** 10.1107/S160053681401277X

**Published:** 2014-06-07

**Authors:** Dominic Kaase, Julia Klingele

**Affiliations:** a Institut für Anorganische und Analytische Chemie, Albert-Ludwigs-Universität Freiburg, Albertstrasse 21, 79104 Freiburg i. Br., Germany

## Abstract

The title compound, [Fe(C_12_H_8_N_4_S)_2_(CH_3_OH)_2_](ClO_4_)_2_, crystallized in the solvent-free form from a methanol solution. The Fe^II^ ion is located on a centre of inversion. The distorted N_4_O_2_ octa­hedral coordination geometry is formed by two *N*,*N*′-chelating equatorial 2,5-bis­(pyridin-2-yl)-1,3,4-thia­diazole ligands and axially coordinating methanol coligands, resulting in the mononuclear *trans*-(*N* ^2^,*N* ^3^,O)_2_ coordination mode. The methanol co-ligand is involved in a hydrogen bond to the perchlorate counter-ion.

## Related literature   

For other 3*d* metal structures of 2,5-bis­(pyridin-2-yl)-1,3,4-thia­diazole, see: Klingele *et al.* (2010 ▶, 2012 ▶); Bentiss, Lagrenee, Mentre *et al.* (2004 ▶); Bentiss, Lagrenee, Vezin *et al.* (2004 ▶); Zheng *et al.* (2006 ▶); Bentiss *et al.* (2002 ▶); Wan *et al.* (2007 ▶). For related compounds, see: Guionneau *et al.* (2004 ▶). For the bridging capability of 4,4′-bispyridine-*N*,*N*′-dioxide, see: Jia *et al.* (2008 ▶).
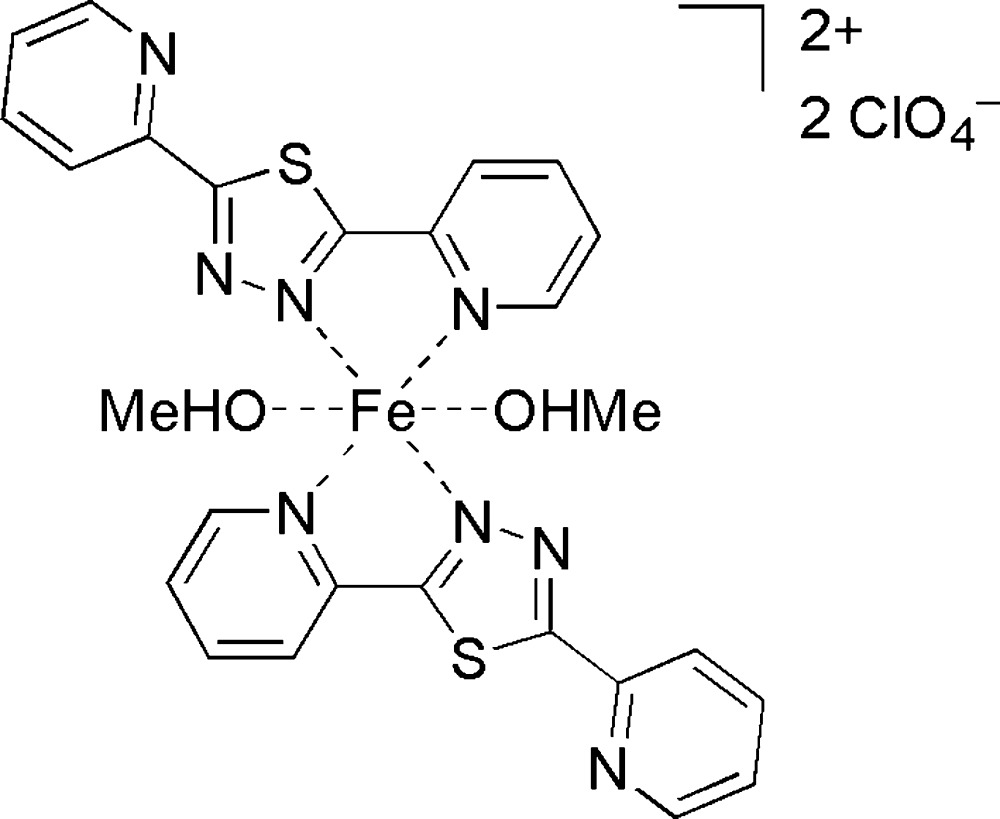


## Experimental   

### 

#### Crystal data  

[Fe(C_12_H_8_N_4_S)_2_(CH_4_O)_2_](ClO_4_)_2_
*M* *_r_* = 799.40
Triclinic, 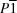

*a* = 8.8410 (3) Å
*b* = 9.5579 (4) Å
*c* = 9.5875 (4) Å
α = 87.169 (2)°
β = 88.945 (2)°
γ = 74.735 (2)°
*V* = 780.61 (5) Å^3^
*Z* = 1
Mo *K*α radiation
μ = 0.86 mm^−1^
*T* = 100 K
0.18 × 0.08 × 0.03 mm

#### Data collection  

Bruker APEXII CCD area-detector diffractometer
Absorption correction: multi-scan (*SADABS*; Bruker, 2001 ▶) *T*_min_ = 0.861, *T*_max_ = 0.975
18025 measured reflections
3128 independent reflections
2789 reflections with *I* > 2σ(*I*)
*R*_int_ = 0.019

#### Refinement  

*R*[*F* ^2^ > 2σ(*F* ^2^)] = 0.027
*wR*(*F* ^2^) = 0.062
*S* = 1.05
3128 reflections
227 parameters
H atoms treated by a mixture of independent and constrained refinement
Δρ_max_ = 0.49 e Å^−3^
Δρ_min_ = −0.38 e Å^−3^

### 

Data collection: *APEX2* (Bruker, 2007 ▶); cell refinement: *SAINT* (Bruker, 2007 ▶); data reduction: *SAINT*; program(s) used to solve structure: *OLEX2.refine* (Puschmann *et al.*, 2013 ▶); program(s) used to refine structure: *SHELXL97* (Sheldrick, 2008 ▶) and *OLEX2.refine* (Puschmann *et al.*, 2013 ▶); molecular graphics: *DIAMOND* (Brandenburg & Putz, 2011 ▶); software used to prepare material for publication: *SHELXL97*.

## Supplementary Material

Crystal structure: contains datablock(s) I. DOI: 10.1107/S160053681401277X/lr2128sup1.cif

Structure factors: contains datablock(s) I. DOI: 10.1107/S160053681401277X/lr2128Isup2.hkl

CCDC reference: 1006207

Additional supporting information:  crystallographic information; 3D view; checkCIF report

## Figures and Tables

**Table 1:** Hydrogen-bond geometry (Å, °)

*D*—H⋯*A*	*D*—H	H⋯*A*	*D*⋯*A*	*D*—H⋯*A*
O20—H20⋯O11	0.78 (3)	1.91 (3)	2.690 (2)	178 (3)

**Fig. 1.**
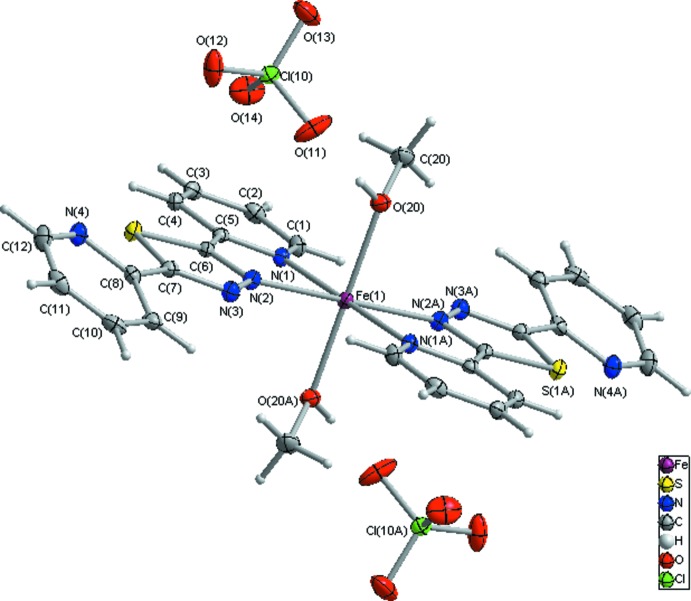
View of the complex [Fe(L)2(MeOH)2](ClO4)2. Displacement ellipsoids are shown at the 50% probability level and H atoms are drawn with arbitrary radii. [Symmetry code: (A) -x, -y+2, -z.]

## supplementary crystallographic information

### S1. Introduction

The ligand 2,5-bis­(pyridin-2-yl)-1,3,4-thia­diazole (L) is well known to have a suitable ligand field for the preparation of iron(II) spin crossover complexes (Klingele. *et al.*, 2010). Known examples are the 2:1-type complexes [Fe(L)_2_(NCS)_2_], [Fe(L)_2_(NCSe)_2_] and [Fe(L)_2_(NCBH_3_)_2_] (Klingele *et al.*, 2012). 4,4'-Bispyridine-*N,N*'-dioxide is able to bridge metal ions to form multidimensional structures (Jia *et al.*, 2008). The title compound [Fe(L)_2_(MeOH)_2_](ClO_4_)_2_ was obtained unintendedly in the attempt to isolate one-dimensional chains of 4,4'-bis­pyridine-*N,N*'-dioxide-bridged [Fe(L)_2_]^2+^ units.

### S2.1. Synthesis and crystallization

Single crystals suitable for X-ray diffraction of the title compound were obtained unexpectedly by layering a MeOH solution of iron(II) perchlorate with a MeOH solution of 2,5-di(pyridin-2-yl)-1,3,4-thia­diazole and 4,4'-bis­pyridine-*N,N*'-dioxide in an argon atmosphere.

### S2.2. Refinement

Crystal data, data collection and structure refinement details are summarized in the Table below. All hydrogen atoms of the ligand and of the methyl hydrogen atoms of the methanol coligand were positioned geometrically and refined using a riding model. The hydrogen atom of the methanol hydroxyl group was located from the difference maps and refined freely and isotropically.

### S3. Results and discussion

The ligand L was synthesized according to literature procedure (Klingele *et al.*, 2012). A single-crystal of [Fe(L)_2_(MeOH)_2_](ClO_4_)_2_ suitable for X-ray diffraction was obtained unexpectedly by layering a MeOH solution of iron(II) perchlorate with a MeOH solution of L and 4,4'-bis­pyridine-*N,N*'-dioxide. The mononuclear complex cation is formed by high-spin iron(II) ion coordinated by two bidentate ligands L and two MeOH coligands. The Fe—N [Fe—N_pyr_ 2.1516 (15), Fe—N_tda_ 2.2015 (15) Å] and Fe—O [2.0886 (13) Å] distances are in the expected range for high-spin iron(II) (Guionneau *et al.*, 2004).

### Crystal data

**Table:**

[Fe(C_12_H_8_N_4_S)_2_(CH_4_O)_2_](ClO_4_)_2_	*Z* = 1
*M**_r_* = 799.40	*F*(000) = 408
Triclinic, *P*1	*D*_x_ = 1.701 Mg m^−^^3^
Hall symbol: -P 1	Mo *K*α radiation, λ = 0.71073 Å
*a* = 8.8410 (3) Å	Cell parameters from 9969 reflections
*b* = 9.5579 (4) Å	θ = 2.2–30.5°
*c* = 9.5875 (4) Å	µ = 0.86 mm^−^^1^
α = 87.169 (2)°	*T* = 100 K
β = 88.945 (2)°	Plate, red
γ = 74.735 (2)°	0.18 × 0.08 × 0.03 mm
*V* = 780.61 (5) Å^3^	

### Data collection

**Table:**

Bruker APEXII CCD area-detector diffractometer	3128 independent reflections
Radiation source: microfocus sealed tube	2789 reflections with *I* > 2σ(*I*)
Multilayer mirror optics monochromator	*R*_int_ = 0.019
φ and ω scans	θ_max_ = 26.4°, θ_min_ = 2.1°
Absorption correction: multi-scan (*SADABS*; Bruker, 2001)	*h* = −11→11
*T*_min_ = 0.861, *T*_max_ = 0.975	*k* = −11→11
18025 measured reflections	*l* = −11→11

### Refinement

**Table:**

Refinement on *F*^2^	Primary atom site location: structure-invariant direct methods
Least-squares matrix: full	Secondary atom site location: difference Fourier map
*R*[*F*^2^ > 2σ(*F*^2^)] = 0.027	Hydrogen site location: inferred from neighbouring sites
*wR*(*F*^2^) = 0.062	H atoms treated by a mixture of independent and constrained refinement
*S* = 1.05	*w* = 1/[σ^2^(*F*_o_^2^) + (0.0172*P*)^2^ + 0.9493*P*] where *P* = (*F*_o_^2^ + 2*F*_c_^2^)/3
3128 reflections	(Δ/σ)_max_ < 0.001
227 parameters	Δρ_max_ = 0.49 e Å^−^^3^
0 restraints	Δρ_min_ = −0.38 e Å^−^^3^

### Special details

**Table:**

Geometry. All e.s.d.'s (except the e.s.d. in the dihedral angle between two l.s. planes) are estimated using the full covariance matrix. The cell e.s.d.'s are taken into account individually in the estimation of e.s.d.'s in distances, angles and torsion angles; correlations between e.s.d.'s in cell parameters are only used when they are defined by crystal symmetry. An approximate (isotropic) treatment of cell e.s.d.'s is used for estimating e.s.d.'s involving l.s. planes.
Refinement. Refinement of *F*^2^ against ALL reflections. The weighted *R*-factor *wR* and goodness of fit *S* are based on *F*^2^, conventional *R*-factors *R* are based on *F*, with *F* set to zero for negative *F*^2^. The threshold expression of *F*^2^ > σ(*F*^2^) is used only for calculating *R*-factors(gt) *etc*. and is not relevant to the choice of reflections for refinement. *R*-factors based on *F*^2^ are statistically about twice as large as those based on *F*, and *R*- factors based on ALL data will be even larger.

### Fractional atomic coordinates and isotropic or equivalent isotropic displacement parameters (Å^2^)

**Table:**

	*x*	*y*	*z*	*U*_iso_*/*U*_eq_	
Fe1	0.0000	1.0000	0.0000	0.01139 (10)	
S1	0.22171 (5)	0.51392 (5)	0.06646 (5)	0.01390 (11)	
N1	0.18464 (17)	0.89318 (16)	−0.14602 (15)	0.0128 (3)	
N2	0.05730 (17)	0.77550 (16)	0.07183 (16)	0.0131 (3)	
N3	−0.00347 (18)	0.70371 (17)	0.17709 (16)	0.0144 (3)	
N4	0.14375 (19)	0.33511 (17)	0.30380 (17)	0.0171 (3)	
C1	0.2469 (2)	0.9547 (2)	−0.25294 (19)	0.0154 (4)	
H1	0.2034	1.0550	−0.2760	0.018*	
C2	0.3724 (2)	0.8783 (2)	−0.33180 (19)	0.0162 (4)	
H2	0.4130	0.9255	−0.4075	0.019*	
C3	0.4371 (2)	0.7330 (2)	−0.2986 (2)	0.0154 (4)	
H3	0.5228	0.6786	−0.3510	0.018*	
C4	0.3748 (2)	0.6677 (2)	−0.18715 (19)	0.0138 (4)	
H4	0.4174	0.5679	−0.1617	0.017*	
C5	0.2495 (2)	0.75062 (19)	−0.11400 (18)	0.0115 (4)	
C6	0.1740 (2)	0.69113 (19)	0.00391 (18)	0.0118 (4)	
C7	0.0705 (2)	0.5668 (2)	0.18660 (19)	0.0131 (4)	
C8	0.0312 (2)	0.4599 (2)	0.28666 (19)	0.0131 (4)	
C9	−0.1131 (2)	0.4898 (2)	0.35465 (19)	0.0158 (4)	
H9	−0.1888	0.5801	0.3387	0.019*	
C10	−0.1429 (2)	0.3835 (2)	0.4465 (2)	0.0186 (4)	
H10	−0.2402	0.3992	0.4949	0.022*	
C11	−0.0285 (2)	0.2540 (2)	0.4665 (2)	0.0200 (4)	
H11	−0.0462	0.1794	0.5288	0.024*	
C12	0.1125 (2)	0.2350 (2)	0.3942 (2)	0.0201 (4)	
H12	0.1908	0.1463	0.4098	0.024*	
C20	−0.2991 (2)	0.9010 (2)	−0.0662 (2)	0.0234 (5)	
H20A	−0.3466	0.8617	−0.1416	0.035*	
H20B	−0.3792	0.9775	−0.0223	0.035*	
H20C	−0.2552	0.8232	0.0036	0.035*	
O20	−0.17589 (15)	0.96075 (15)	−0.12275 (15)	0.0173 (3)	
H20	−0.212 (3)	1.021 (3)	−0.179 (3)	0.026*	
Cl10	−0.46099 (5)	1.25579 (5)	−0.29619 (5)	0.01521 (11)	
O11	−0.30426 (17)	1.16340 (19)	−0.32171 (16)	0.0353 (4)	
O12	−0.47275 (19)	1.39590 (16)	−0.36152 (17)	0.0320 (4)	
O13	−0.57261 (18)	1.19384 (18)	−0.35684 (18)	0.0339 (4)	
O14	−0.48818 (18)	1.26721 (19)	−0.14843 (15)	0.0327 (4)	

### Atomic displacement parameters (Å^2^)

**Table:**

	*U*^11^	*U*^22^	*U*^33^	*U*^12^	*U*^13^	*U*^23^
Fe1	0.01072 (18)	0.00969 (19)	0.01282 (19)	−0.00126 (14)	0.00130 (14)	0.00030 (14)
S1	0.0141 (2)	0.0101 (2)	0.0162 (2)	−0.00128 (17)	0.00275 (17)	0.00076 (18)
N1	0.0137 (8)	0.0124 (8)	0.0118 (8)	−0.0027 (6)	0.0007 (6)	−0.0007 (6)
N2	0.0133 (7)	0.0125 (8)	0.0128 (8)	−0.0024 (6)	0.0018 (6)	0.0005 (6)
N3	0.0159 (8)	0.0134 (8)	0.0136 (8)	−0.0042 (6)	0.0017 (6)	0.0012 (6)
N4	0.0202 (8)	0.0136 (8)	0.0171 (8)	−0.0041 (7)	0.0023 (7)	0.0009 (7)
C1	0.0184 (9)	0.0129 (9)	0.0153 (9)	−0.0048 (8)	0.0005 (7)	−0.0001 (7)
C2	0.0177 (9)	0.0198 (10)	0.0134 (9)	−0.0090 (8)	0.0025 (7)	−0.0020 (8)
C3	0.0124 (9)	0.0184 (10)	0.0163 (9)	−0.0047 (7)	0.0026 (7)	−0.0070 (8)
C4	0.0137 (9)	0.0115 (9)	0.0157 (9)	−0.0021 (7)	−0.0010 (7)	−0.0026 (7)
C5	0.0119 (8)	0.0128 (9)	0.0104 (8)	−0.0041 (7)	−0.0022 (7)	−0.0014 (7)
C6	0.0117 (8)	0.0109 (9)	0.0126 (9)	−0.0026 (7)	−0.0025 (7)	−0.0009 (7)
C7	0.0115 (9)	0.0152 (9)	0.0127 (9)	−0.0036 (7)	−0.0007 (7)	−0.0015 (7)
C8	0.0159 (9)	0.0129 (9)	0.0118 (9)	−0.0060 (7)	−0.0007 (7)	−0.0015 (7)
C9	0.0152 (9)	0.0170 (10)	0.0161 (9)	−0.0057 (8)	−0.0035 (7)	−0.0003 (8)
C10	0.0184 (10)	0.0262 (11)	0.0153 (10)	−0.0130 (8)	0.0002 (8)	−0.0026 (8)
C11	0.0301 (11)	0.0198 (10)	0.0144 (10)	−0.0145 (9)	0.0005 (8)	0.0011 (8)
C12	0.0272 (11)	0.0131 (10)	0.0189 (10)	−0.0037 (8)	−0.0001 (8)	0.0015 (8)
C20	0.0144 (10)	0.0255 (11)	0.0320 (12)	−0.0074 (8)	0.0032 (8)	−0.0068 (9)
O20	0.0145 (7)	0.0178 (7)	0.0192 (7)	−0.0037 (6)	−0.0026 (5)	0.0009 (6)
Cl10	0.0138 (2)	0.0166 (2)	0.0151 (2)	−0.00390 (17)	0.00130 (17)	0.00016 (18)
O11	0.0177 (8)	0.0490 (11)	0.0267 (9)	0.0102 (7)	0.0051 (6)	0.0129 (8)
O12	0.0452 (10)	0.0162 (8)	0.0349 (9)	−0.0095 (7)	0.0194 (7)	−0.0019 (7)
O13	0.0289 (9)	0.0383 (10)	0.0423 (10)	−0.0201 (7)	0.0035 (7)	−0.0160 (8)
O14	0.0312 (9)	0.0527 (11)	0.0135 (7)	−0.0105 (8)	0.0059 (6)	−0.0004 (7)

### Geometric parameters (Å, º)

**Table:**

Fe1—O20	2.0886 (13)	C1—C2	1.391 (3)
Fe1—O20^i^	2.0886 (13)	C2—C3	1.379 (3)
Fe1—N2^i^	2.1516 (15)	C3—C4	1.389 (3)
Fe1—N2	2.1516 (15)	C4—C5	1.382 (3)
Fe1—N1^i^	2.2015 (15)	C5—C6	1.468 (2)
Fe1—N1	2.2015 (15)	C7—C8	1.471 (2)
S1—C6	1.7144 (18)	C8—C9	1.389 (3)
S1—C7	1.7364 (19)	C9—C10	1.386 (3)
N1—C1	1.338 (2)	C10—C11	1.384 (3)
N1—C5	1.354 (2)	C11—C12	1.389 (3)
N2—C6	1.315 (2)	C20—O20	1.443 (2)
N2—N3	1.372 (2)	Cl10—O13	1.4232 (15)
N3—C7	1.299 (2)	Cl10—O12	1.4292 (15)
N4—C12	1.339 (2)	Cl10—O14	1.4372 (15)
N4—C8	1.342 (2)	Cl10—O11	1.4574 (15)
			
O20—Fe1—O20^i^	180.00 (4)	C2—C3—C4	118.88 (17)
O20—Fe1—N2^i^	91.48 (6)	C5—C4—C3	118.73 (17)
O20^i^—Fe1—N2^i^	88.52 (6)	N1—C5—C4	122.90 (16)
O20—Fe1—N2	88.52 (6)	N1—C5—C6	114.25 (16)
O20^i^—Fe1—N2	91.48 (6)	C4—C5—C6	122.85 (16)
N2^i^—Fe1—N2	180.0	N2—C6—C5	120.39 (16)
O20—Fe1—N1^i^	87.99 (5)	N2—C6—S1	113.48 (13)
O20^i^—Fe1—N1^i^	92.01 (5)	C5—C6—S1	126.13 (14)
N2^i^—Fe1—N1^i^	76.37 (6)	N3—C7—C8	124.64 (17)
N2—Fe1—N1^i^	103.63 (6)	N3—C7—S1	114.73 (13)
O20—Fe1—N1	92.01 (5)	C8—C7—S1	120.61 (14)
O20^i^—Fe1—N1	87.99 (5)	N4—C8—C9	124.49 (17)
N2^i^—Fe1—N1	103.63 (6)	N4—C8—C7	114.70 (16)
N2—Fe1—N1	76.37 (6)	C9—C8—C7	120.81 (17)
N1^i^—Fe1—N1	180.0	C10—C9—C8	117.74 (18)
C6—S1—C7	86.84 (9)	C11—C10—C9	118.93 (18)
C1—N1—C5	117.61 (16)	C10—C11—C12	118.91 (18)
C1—N1—Fe1	127.76 (12)	N4—C12—C11	123.43 (19)
C5—N1—Fe1	114.39 (11)	C20—O20—Fe1	123.03 (12)
C6—N2—N3	113.63 (15)	O13—Cl10—O12	109.10 (10)
C6—N2—Fe1	114.27 (12)	O13—Cl10—O14	110.30 (10)
N3—N2—Fe1	132.09 (12)	O12—Cl10—O14	110.33 (10)
C7—N3—N2	111.33 (15)	O13—Cl10—O11	108.75 (10)
C12—N4—C8	116.48 (17)	O12—Cl10—O11	108.62 (9)
N1—C1—C2	122.82 (17)	O14—Cl10—O11	109.71 (9)
C3—C2—C1	119.05 (17)		
			
O20—Fe1—N1—C1	92.67 (15)	Fe1—N2—C6—C5	−2.5 (2)
O20^i^—Fe1—N1—C1	−87.33 (15)	N3—N2—C6—S1	−0.96 (19)
N2^i^—Fe1—N1—C1	0.65 (16)	Fe1—N2—C6—S1	178.04 (8)
N2—Fe1—N1—C1	−179.35 (16)	N1—C5—C6—N2	−2.2 (2)
O20—Fe1—N1—C5	−93.28 (12)	C4—C5—C6—N2	178.55 (16)
O20^i^—Fe1—N1—C5	86.72 (12)	N1—C5—C6—S1	177.18 (13)
N2^i^—Fe1—N1—C5	174.71 (12)	C4—C5—C6—S1	−2.0 (3)
N2—Fe1—N1—C5	−5.29 (12)	C7—S1—C6—N2	0.74 (14)
O20—Fe1—N2—C6	96.46 (13)	C7—S1—C6—C5	−178.71 (16)
O20^i^—Fe1—N2—C6	−83.54 (13)	N2—N3—C7—C8	−178.46 (16)
N1^i^—Fe1—N2—C6	−175.97 (12)	N2—N3—C7—S1	−0.07 (19)
N1—Fe1—N2—C6	4.03 (12)	C6—S1—C7—N3	−0.37 (15)
O20—Fe1—N2—N3	−84.78 (15)	C6—S1—C7—C8	178.09 (15)
O20^i^—Fe1—N2—N3	95.22 (15)	C12—N4—C8—C9	−0.7 (3)
N1^i^—Fe1—N2—N3	2.79 (16)	C12—N4—C8—C7	179.92 (17)
N1—Fe1—N2—N3	−177.21 (16)	N3—C7—C8—N4	−163.82 (17)
C6—N2—N3—C7	0.7 (2)	S1—C7—C8—N4	17.9 (2)
Fe1—N2—N3—C7	−178.11 (13)	N3—C7—C8—C9	16.8 (3)
C5—N1—C1—C2	0.7 (3)	S1—C7—C8—C9	−161.50 (14)
Fe1—N1—C1—C2	174.61 (13)	N4—C8—C9—C10	0.0 (3)
N1—C1—C2—C3	−0.5 (3)	C7—C8—C9—C10	179.33 (17)
C1—C2—C3—C4	−0.1 (3)	C8—C9—C10—C11	0.3 (3)
C2—C3—C4—C5	0.3 (3)	C9—C10—C11—C12	0.1 (3)
C1—N1—C5—C4	−0.4 (3)	C8—N4—C12—C11	1.2 (3)
Fe1—N1—C5—C4	−175.14 (13)	C10—C11—C12—N4	−0.9 (3)
C1—N1—C5—C6	−179.67 (15)	N2^i^—Fe1—O20—C20	−124.06 (14)
Fe1—N1—C5—C6	5.64 (19)	N2—Fe1—O20—C20	55.94 (14)
C3—C4—C5—N1	−0.1 (3)	N1^i^—Fe1—O20—C20	−47.76 (14)
C3—C4—C5—C6	179.08 (16)	N1—Fe1—O20—C20	132.24 (14)
N3—N2—C6—C5	178.53 (15)		

Symmetry code: (i) −*x*, −*y*+2, −*z*.

### Hydrogen-bond geometry (Å, º)

**Table:**

*D*—H···*A*	*D*—H	H···*A*	*D*···*A*	*D*—H···*A*
O20—H20···O11	0.78 (3)	1.91 (3)	2.690 (2)	178 (3)
